# Supplementary material for: Advanced three-dimensional X-ray imaging unravels structural development of the human thymus compartments
Source: Commun Med (Lond). 2024 Oct 22;4:204. doi: 10.1038/s43856-024-00623-7 (PMC11496816; doi:10.1038/s43856-024-00623-7)
Supplement: Supplementary file 2 — Supplementary Information [file 43856_2024_623_MOESM2_ESM.pdf]

Supplementary Information to:

Advanced three-dimensional X-ray imaging unravels structural development of the human thymus compartments

Savvas Savvidis[1], Roberta Ragazzini[2,3], Valeria Conde de Rafael[1,2,3], J. Ciaran Hutchinson[4], Lorenzo Massimi[1], Fabio A. Vittoria[1‡], Sara Campinoti[2,3¥], Tom Partridge[1], Olumide K. Ogunbiyi[4], Alessia Atzeni[5], Neil J. Sebire[4], Paolo De Coppi[6,7], Alberto Mittone[8†], Alberto Bravin[8,9], Paola Bonfanti[2,3*] and Alessandro Olivo[1*]

[1]Department of Medical Physics and Biomedical Engineering, University College London, London, WC1E 6BT, UK.

[2]Epithelial Stem Cell Biology & Regenerative Medicine laboratory, The Francis Crick Institute, London NW1 1AT, UK.

[3]Institute of Immunity & Transplantation, Division of Infection & Immunity, UCL, London NW3 2PP, UK.

[4]Department of Histopathology, Great Ormond Street Hospital for Children NHS Foundation Trust, London WC1N 1EH, UK.

[5]Centre for Medical Image Computing, Department of Medical Physics and Biomedical Engineering, University College London, WC1E 6BT, UK.

[6]Stem Cell and Regenerative Medicine Section, Great Ormond Street Institute of Child Health, University College London, London WC1N 1EH, UK.

Specialist Neonatal and Paediatric Surgery, Great Ormond Street Hospital NHS Trust.

[8]European Synchrotron Radiation Facility, Grenoble, 38043, France.

[9]University Milano Bicocca, Dept. of Physics "G. Occhialini", Milano, Italy.

[‡]current address: ENEA - Radiation Protection Institute, Via Martiri di Monte Sole 4, 40129 Bologna, Italy

[¥]current address: The Roger Williams Institute of Hepatology, 111 Coldharbour Lane, SE5 9NT London, UK

[†]current address: Advanced Photon Source, Argonne National Labs, Lemont, IL

*Corresponding authors: A.O. (a.olivo@ucl.ac.uk) and P.B. (paola.bonfanti@crick.ac.uk). These authors jointly supervised this work.

Supplementary Figure 1. Visualisation of Hassall's bodies at gestation week 19.

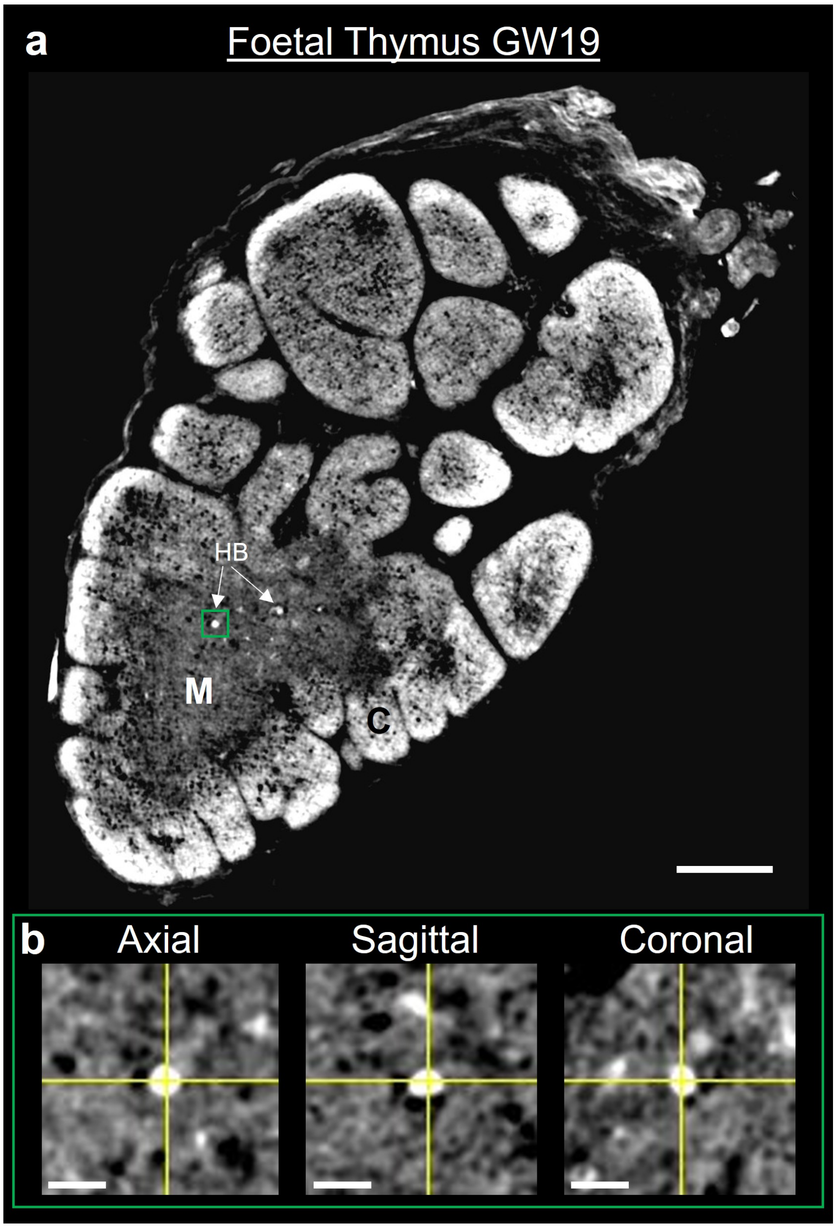

a Hassall's bodies (HB) visualisation in foetal thymus at gestation week (GW) 19. The HB indicated in the green box in **a** is also shown in all anatomical planes in **b**. Scale bars: 500 µm in **a**, 100 µm in **b.**

Supplementary Figure 2. Segmentation of cortex and medulla in SPC-CT and Histological H&E slices.

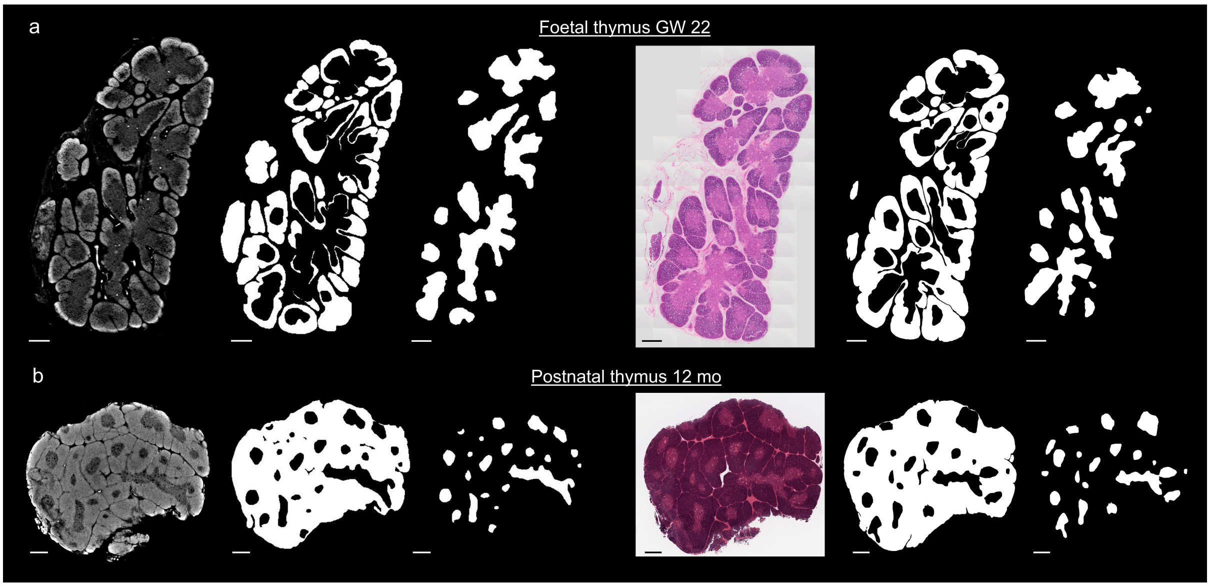

The SPC-CT slices and Histological (H&E) images of the foetal (**a**) and postnatal (**b**) thymi shown in Fig. 2 of the main article are presented along with their corresponding cortex (left) and medulla (right) binary masks. These masks were used for generating the results reported in supplementary Table 1, by applying the approach to extract the volumetric medulla content described in the methods to a single slice. Scale bars: 500 µm.

Supplementary Figure 3. Medulla content extracted from entire organs and from the central 300 SPC-CT slices.

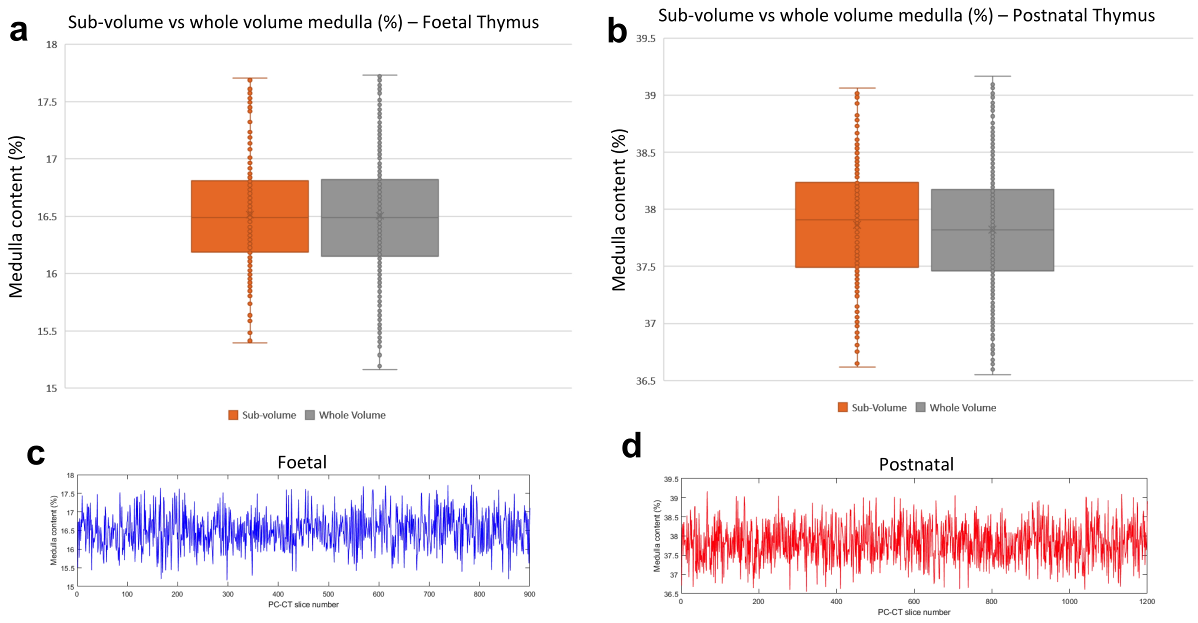

Box plots in (**a**) and (**b**) show the medulla content extracted from the central 300 slices (orange) and full organ (grey) for a foetal and postnatal thymus, respectively. Full volumes correspond to 900 and 1200 slices in the two cases. (**c**) and (**d**) show numerical results for all slices in the foetal and postnatal thymus, respectively.

Supplementary Figure 4. Representative gating strategy for flow-cytometry analysis of human foetal (GW 15) and postnatal (4 MPN) samples.

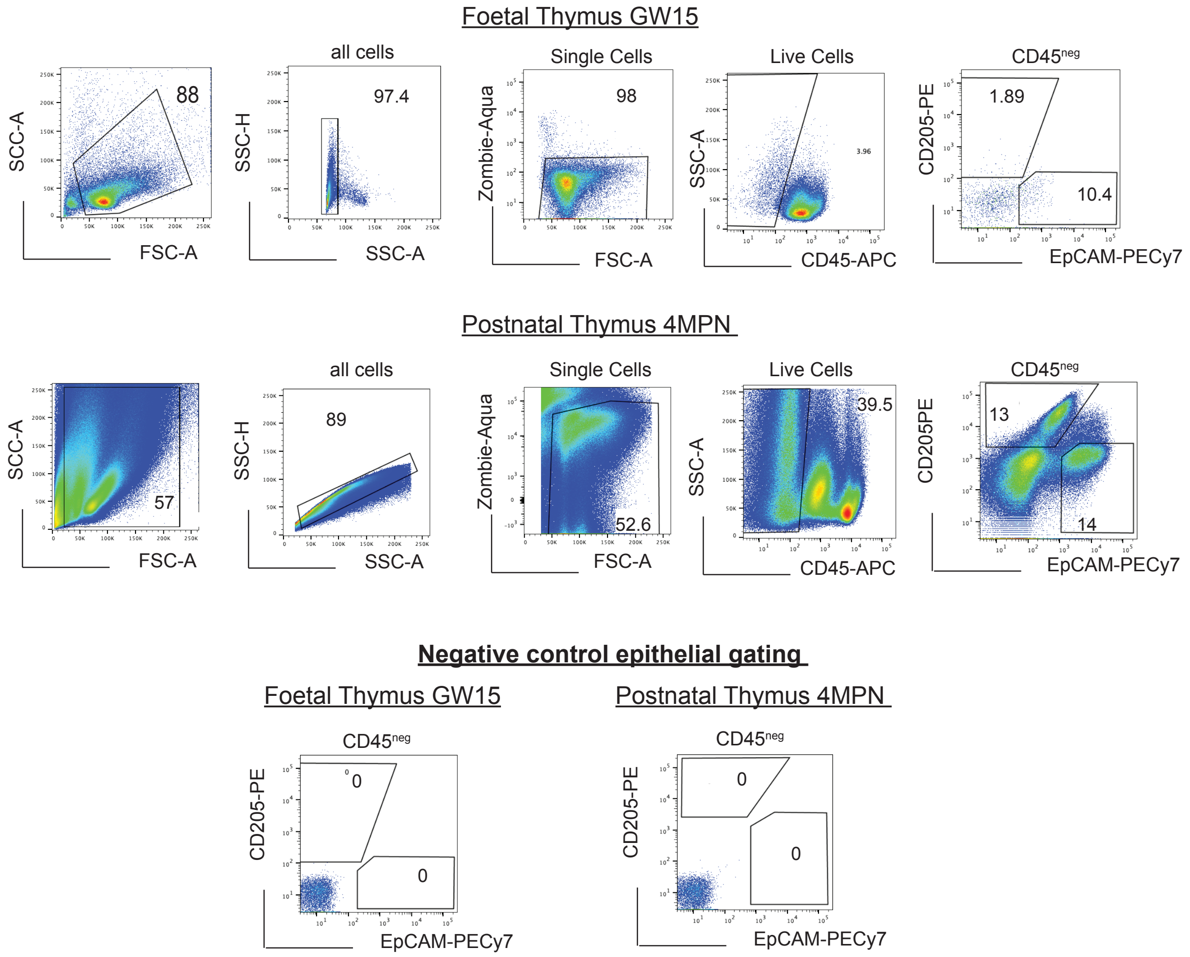

Graphs show total cell population, single cell, live cells, and non-immune fraction (CD45negative). Negative controls are represented by the same samples stained only with live/dead (Zombie-Aqua as viability dye).

Supplementary Figure 5. HB content quantification on 2D histological sections.

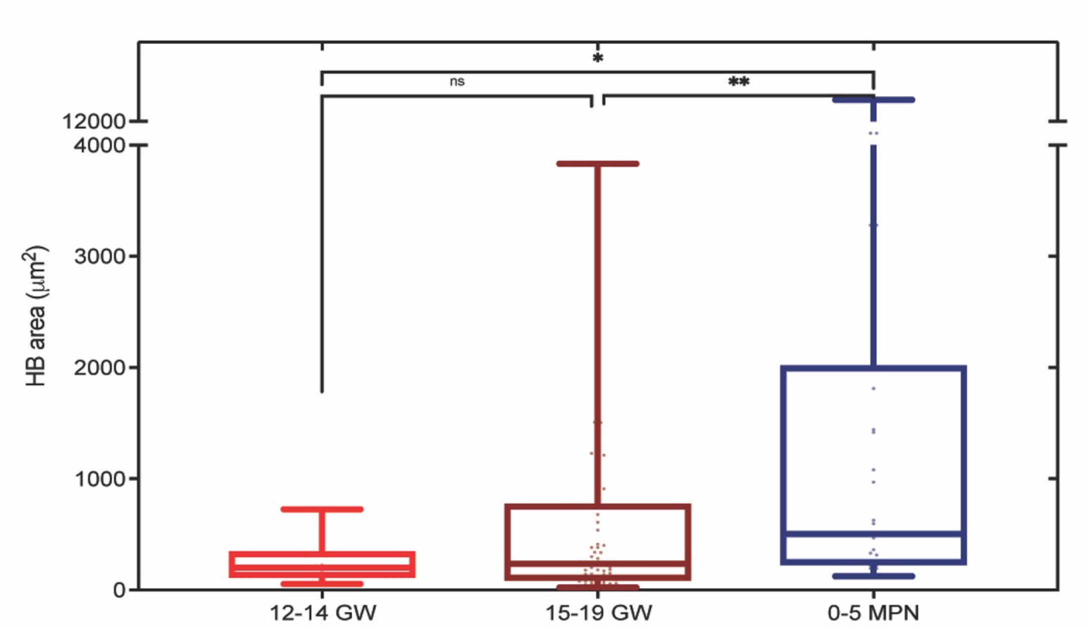

Machine learning-based quantification of relative medullary area across three developmental phases in H&E slices (n=5 per group, with n=3 technical triplicates). Earlier stage samples could not be assessed since HBs were not detected in the H&E slices.

Supplementary Figure 6. Segmentation of cortex and medulla in H-CT scans.

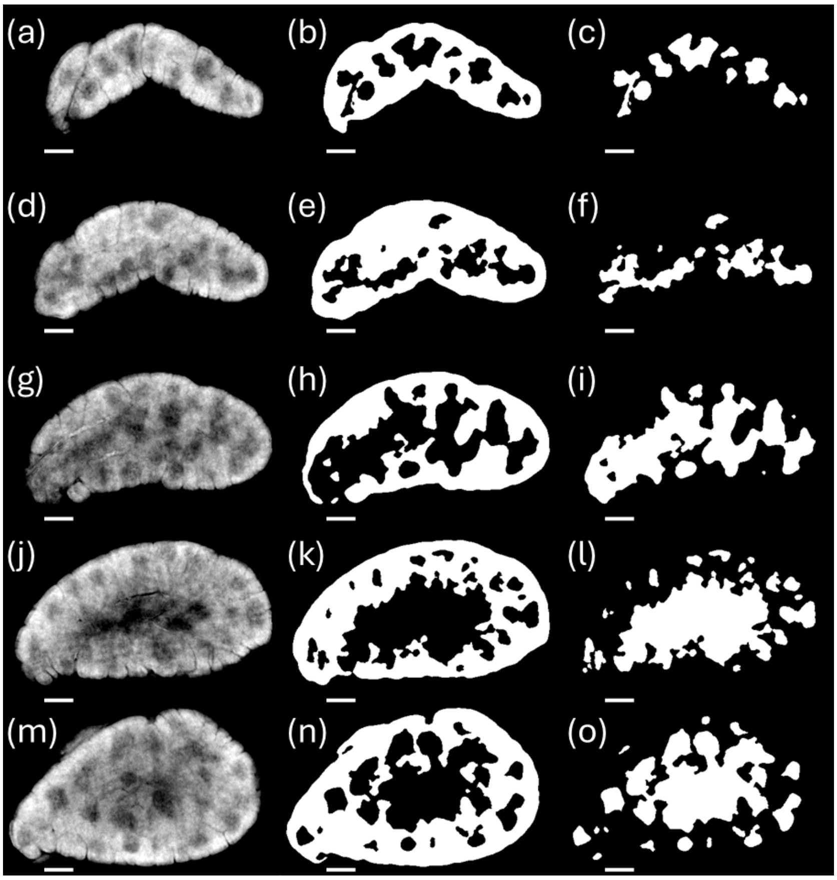

A 19-day post-natal thymus is shown as an example**.** (a), (d), (g), (j), and (m) show the H-CT slices, while (b), (e), (h), (k), and (n) and (c), (f), (i), (l), and (o) show the associated segmented cortex and medulla regions, respectively. Top and bottom slices (a) and (m) were segmented manually to support the automated segmentation of the intermediate slices, among which (non-adjacent) slices (d), (g), and (j). The slices were window averaged (5 slices) and median filtered prior to segmentation using the trainable WEKA fast random forest algorithm implemented in Fiji (see methods section). Scale bars: 500 μm.

Supplementary Table 1. Medulla content for SPC-CT and corresponding Histological H&E slices obtained from the binary masks shown in Supplementary Figure 2. As can be seen, a good quantitative agreement is obtained for the postnatal and foetal sample, respectively. One should note that these values are also affected by the manual approach adapted to match SPC-CT and H&E slices. The validation of the quantitative medulla content extraction supports the use of the proposed PC-CT based volumetric quantification, which is not affected by loss of tissue through sectioning and allows analysing the entire sample as opposed to only a fraction, thereby leading to a more accurate quantification of structural changes in different contexts.

**Sample**	**Medulla Content (%) single slice**	
	SPC-CT Slice	Histology (H&E)
GW22	37.9	34.6
12mo	16.5	16.4

Supplementary Table 2. Numerical values for medulla content extracted from the entire organ and from the central 300 slices for examples of foetal and postnatal thymi (see Supp. Fig. 3).

	Postnatal whole (slices 1-1200)	Postnatal sub-volume (slices 500-800)	Foetal whole (slices 1-900)	Foetal sub-volume (slices 350-650)
Mean	37.82	37.86	16.50	16.49
Median	37.82	37.91	16.49	16.51
stdv	0.50	0.49	0.49	0.46
max	39.62	39.55	17.73	17.71
min	39.06	39.17	15.16	15.39

Equations used to quantify Medulla and Hassall Body content

Equation S1.1: Volumetric Medulla Content =

$$\frac{\sum voxels_{\ Volume\ of\ medulla\ masks}}{(\sum voxels_{Volume\ of\ cortex\ masks} + \sum voxels_{\ Volume\ of\ medulla\ masks})} \times 100$$

Equation S1.2: Volumetric HBs Content =

$$\frac{\sum voxels_{\ Volume\ of\ HB\ masks}}{(\sum voxels_{Volume\ of\ medulla\ masks})} \times 100$$

Equation S1.3: Histology-based Medulla Content =

$$\frac{\sum pixels_{medulla}}{(\sum pixels_{medulla} + \sum pixels_{cortex})} \times 100$$

Equation S1.4: FACS-based Medulla Content =

$$\frac{\sum voxels_{medullary\ cell\ percentage}}{(\sum voxels_{medullry\ cell\ percentage} + \sum voxels_{cortical\ cell\ percentage})} \times 100$$
