# Supplementary material for: Advanced three-dimensional X-ray imaging unravels structural development of the human thymus compartments
Source: Commun Med (Lond). 2024 Oct 22;4:204. doi: 10.1038/s43856-024-00623-7 (PMC11496816; doi:10.1038/s43856-024-00623-7)
Supplement: Supplementary file 3 — Description of Additional Supplementary Files [file 43856_2024_623_MOESM3_ESM.pdf]

Description of Additional Supplementary Files

File name: Supplementary Data 1

File description: Data for graphs presented in Fig 4

File name: Supplementary Data 2

File description: Data for graph presented in Fig 5
