# Supplementary material for: Advanced three-dimensional X-ray imaging unravels structural development of the human thymus compartments
Source: Commun Med (Lond). 2024 Oct 22;4:204. doi: 10.1038/s43856-024-00623-7 (PMC11496816; doi:10.1038/s43856-024-00623-7)
Supplement: Supplementary file 6 — Reporting Summary [file 43856_2024_623_MOESM6_ESM.pdf]

nature portfolio

Corresponding author(s): Alessandro Olivo and Paola Bonfanti

Last updated by author(s): Feb 15, 2024

Reporting Summary

Nature Portfolio wishes to improve the reproducibility of the work that we publish. This form provides structure for consistency and transparency in reporting. For further information on Nature Portfolio policies, see our Editorial Policies and the Editorial Policy Checklist.

Statistics

For all statistical analyses, confirm that the following items are present in the figure legend, table legend, main text, or Methods section.

n/a	Confirmed	
☐	☒	The exact sample size (*n*) for each experimental group/condition, given as a discrete number and unit of measurement
☐	☒	A statement on whether measurements were taken from distinct samples or whether the same sample was measured repeatedly
☐	☒	The statistical test(s) used AND whether they are one- or two-sided<br>*Only common tests should be described solely by name; describe more complex techniques in the Methods section.*
☒	☐	A description of all covariates tested
☒	☐	A description of any assumptions or corrections, such as tests of normality and adjustment for multiple comparisons
☒	☐	A full description of the statistical parameters including central tendency (e.g. means) or other basic estimates (e.g. regression coefficient) AND variation (e.g. standard deviation) or associated estimates of uncertainty (e.g. confidence intervals)
☒	☐	For null hypothesis testing, the test statistic (e.g. *F*, *t*, *r*) with confidence intervals, effect sizes, degrees of freedom and *P* value noted<br>*Give P values as exact values whenever suitable.*
☒	☐	For Bayesian analysis, information on the choice of priors and Markov chain Monte Carlo settings
☒	☐	For hierarchical and complex designs, identification of the appropriate level for tests and full reporting of outcomes
☒	☐	Estimates of effect sizes (e.g. Cohen's *d*, Pearson's *r*), indicating how they were calculated

Our web collection on statistics for biologists contains articles on many of the points above.

Software and code

Policy information about availability of computer code

Data collection	Not applicable (custom code developed by beamline ID17 scientists at the ESRF and by the UCL AXIm group for the lab scans)
Data analysis	Not applicable, analysis software was custom-written along the lines described in the manuscript. Fiji and Drishti were used for image visualization, this is also specified.

For manuscripts utilizing custom algorithms or software that are central to the research but not yet described in published literature, software must be made available to editors and reviewers. We strongly encourage code deposition in a community repository (e.g. GitHub). See the Nature Portfolio guidelines for submitting code & software for further information.

Data

Policy information about availability of data

All manuscripts must include a data availability statement. This statement should provide the following information, where applicable:

- Accession codes, unique identifiers, or web links for publicly available datasets
- A description of any restrictions on data availability
- For clinical datasets or third party data, please ensure that the statement adheres to our policy

Data and code are available from the corresponding authors

Research involving human participants, their data, or biological material

Policy information about studies with human participants or human data. See also policy information about sex, gender (identity/presentation), and sexual orientation and race, ethnicity and racism.

Reporting on sex and gender	This study used anonymized tissue, so none of these questions are relevant.
Reporting on race, ethnicity, or other socially relevant groupings	N.A.
Population characteristics	N.A.
Recruitment	N.A.
Ethics oversight	Research Tissue Bank ethical approval (REC No 08/H0712/34+5 and 08/H0906/21+5; REC No 15/YH/0334)

Note that full information on the approval of the study protocol must also be provided in the manuscript.

Field-specific reporting

Please select the one below that is the best fit for your research. If you are not sure, read the appropriate sections before making your selection.

☒ Life sciences ☐ Behavioural & social sciences ☐ Ecological, evolutionary & environmental sciences

For a reference copy of the document with all sections, see nature.com/documents/nr-reporting-summary-flat.pdf

Life sciences study design

All studies must disclose on these points even when the disclosure is negative.

Sample size	15 thymi
Data exclusions	N.A.
Replication	N.A.
Randomization	N.A.
Blinding	N.A.

Reporting for specific materials, systems and methods

We require information from authors about some types of materials, experimental systems and methods used in many studies. Here, indicate whether each material, system or method listed is relevant to your study. If you are not sure if a list item applies to your research, read the appropriate section before selecting a response.

Materials & experimental systems

n/a	Involved in the study	
☐	☒	Antibodies
☒	☐	Eukaryotic cell lines
☒	☐	Palaeontology and archaeology
☒	☐	Animals and other organisms
☒	☐	Clinical data
☒	☐	Dual use research of concern
☒	☐	Plants

Methods

n/a	Involved in the study	
☒	☐	ChIP-seq
☐	☒	Flow cytometry
☒	☐	MRI-based neuroimaging

Antibodies

Antibodies used	See table 1 in manuscript for full list
Validation	-FACS antibodies purchased from Biolegends have been validated according to manufacturer's instructions: https://www.biolegend.com/nl-nl/reproducibility

Primary antibody validation:
REAGENT or RESOURCE SOURCE IDENTIFIER:
- KRT10 mouse antibody 1:200; Santa-Cruz SC-53252; RRID:AB_629 835

Plants

Seed stocks	N.A.
Novel plant genotypes	N.A.
Authentication	N.A.

Flow Cytometry

Plots

Confirm that:

☒ The axis labels state the marker and fluorochrome used (e.g. CD4-FITC).

☒ The axis scales are clearly visible. Include numbers along axes only for bottom left plot of group (a 'group' is an analysis of identical markers).

☒ All plots are contour plots with outliers or pseudocolor plots.

☒ A numerical value for number of cells or percentage (with statistics) is provided.

Methodology

Sample preparation	Thymic tissues were dissociated to single cell suspension with enzymatic treatment (Collagenase-Dispase) for around 30-45 minutes, using the Gentle MACS machine (Miltenyi). After the dissociation, the supernatant was collected, passed through a cell-strainer, centrifuged for 5 minutes and counted with trypan blue (SIGMA-ALDRICH) to assess viability. See further details in Material and Methods.
Instrument	Fortessa X-20 machine (BD Bioscience)
Software	BD FACS-Diva abd FlowJoTM software.
Cell population abundance	Cell population abundance is specified in the figure legends
Gating strategy	Cells were first gated based on light scatter properties (FSC-A) and granularity (SSC-A), and doublets were excluded by gating SSC Width against Hight. Dead cells were excluded on the basis of their positivity or Live/Dead markers (Zombie, biolegend). Cells expressing relevant markers were classified on unstained negative cells or L/D only staining control.

☒ Tick this box to confirm that a figure exemplifying the gating strategy is provided in the Supplementary Information.
